# The Hydrogen Sulfide Releasing Molecule Acetyl Deacylasadisulfide Inhibits Metastatic Melanoma

**DOI:** 10.3389/fphar.2017.00065

**Published:** 2017-02-27

**Authors:** Paola De Cicco, Elisabetta Panza, Chiara Armogida, Giuseppe Ercolano, Orazio Taglialatela-Scafati, Yalda Shokoohinia, Rosa Camerlingo, Giuseppe Pirozzi, Vincenzo Calderone, Giuseppe Cirino, Angela Ianaro

**Affiliations:** ^1^Department of Pharmacy, University of Naples Federico IINaples, Italy; ^2^Department of Pharmacognosy and Biotechnology, School of Pharmacy, Kermanshah University of Medical SciencesKermanshah, Iran; ^3^Department of Experimental Oncology, National Cancer Institute -IRCCS “G.Pascale” FoundationNaples, Italy; ^4^Department of Pharmacy, University of PisaPisa, Italy

**Keywords:** melanoma, skin cancer, hydrogen sulfide, apoptosis, metastasis

## Abstract

Melanoma is the most common form of skin cancer. Given its high mortality, the interest in the search of preventive measures, such as dietary factors, is growing significantly. In this study we tested, *in vitro* and *in vivo*, the potential anti-cancer effect of the acetyl deacylasadisulfide (ADA), a vinyl disulfide compound, isolated and purified from asafoetida a foul-smelling oleo gum-resin of dietary and medicinal relevance. ADA markedly suppressed proliferation of human melanoma cell lines by inducing apoptosis. Moreover, treatment of melanoma cells with ADA reduced nuclear translocation and activation of NF-κB, decreased the expression of the anti-apoptotic proteins c-FLIP, XIAP, and Bcl-2 and inhibited the phosphorylation and activation of both AKT and ERK proteins, two of the most frequently deregulated pathways in melanoma. Finally, the results obtained *in vitro* were substantiated by the findings that ADA significantly and dose-dependently reduced lung metastatic foci formation in C57BL/6 mice. In conclusion, our findings suggest that ADA significantly inhibits melanoma progression *in vivo* and could represent an important lead compound for the development of new anti-metastatic agents.

## Introduction

Hydrogen sulfide (H_2_S) is a gaseous signaling molecule that plays important roles in a variety of biological functions, in health and disease (Szabo, 2007; Kimura, 2011; Whiteman et al., 2011; Wang, 2012). The physiological production of H_2_S is mainly deputed to the activity of three enzymes: Cystathionine-β-synthase (CBS), cystathionine-γ-lyase (CSE), and 3-mercaptopyruvate sulfurtransferase (3-MST). Recently endogenous generated H_2_S has been involved in the regulation of cancer biological processes and, according to the cancer type, different roles can be ascribed to the molecule as recently reviewed (Szabo, 2016). In colorectal cancer, ovarian cancer, and breast cancer an increase in the expression of CBS in cancer cells has been associated to the promotion of cell proliferation and cellular bioenergetics (Hellmich and Szabo, 2015). In human melanoma, H_2_S generated by over-expressed CSE appears to be involved in the progression of the disease (Panza et al., 2015). While inhibition of H_2_S biosynthesis produces anticancer effects, many reports show that H_2_S donors, of either natural or chemical origin, exert anticancer actions *in vitro* and *in vivo* (Hellmich et al., 2015; Panza et al., 2015). Thus, it has been hypothesized that low (endogenous) levels of H_2_S tend to promote, while higher levels released from donors (exogenous), tend to inhibit cancer cell proliferation (Hellmich et al., 2015).

*Ferula assa-foetida* is the main source of asafoetida, an oleo-gum resin obtained by incision of the roots of various plants from the genus *Ferula* (family Umbelliferae) native to Central Asia, Afghanistan and Iran (Mahendra and Bisht, 2012). Asafoetida chiefly subsume resin (40–65%), gum (20–25%), and volatile oil (3–17%) the latter consisting of disulfides as its major components that are responsible for the characteristic odor of asafoetida (Iranshahy and Iranshahi, 2011). Asafoetida is a popular ingredient in the Indian cuisine and it is also used in traditional medicine for treating many human diseases such as asthma, gastro-intestinal disorders, influenza, and more recently, a cancer chemopreventive role for asafoetida has been described (Kim et al., 2011; Kiani et al., 2015; Oh et al., 2015). Different mechanisms seem to impact on this activity such as radical scavenging activity of sulfur-containing compounds even if the exact mechanism through which asafoetida behaves as ant-tumor agent has yet to be elucidated. In the present study, we have isolated and purified from asafoetida a new H_2_S-donor, *ADA*, studied and clarified its mechanism of action *in vitro* and demonstrated its anti-tumoral activity *in vivo*.

## Methods

### Plant material and isolation of vinyl disulfides

The latex of *F. asafoetida* was collected by incision of the root collar from plants growing in Jandagh (Isfahan, Iran) at an altitude of 1500 m above sea level. The plant material was identified by Dr. Mohammad-Reza Kanani, Department of Biology, Medicinal Plants and Drugs Research Institute, Shahid Beheshti University, Tehran, Iran, where a voucher specimen is kept (School of Pharmacy, Isfahan, No. 5729). The latex (100 g) was dried and extracted with acetone (2 × 1 L) for 2 days with continuous shaking to afford a gummy residue (30 g), that was fractionated by gravity column chromatography on silica gel and further purified by HPLC to obtain pure compounds as previously described (Shokoohinia et al., 2013).

### Reagents and cell culture

Normal human epidermal melanocytes (NHEM) were purchased from Lonza (Walkersville, MD, USA) and were grown in Melanocyte growth medium 2 (Lonza). The melanoma cells lines B16/F10, Sk-Mel-5, and Sk-Mel-28 were purchased from IRCCS AOU San Martino—IST (Genova, Italy), A375 from Sigma-Aldrich (Milan, Italy), and WM3060 and WM983A were purchased from Rockland (Limerick, Ireland). B16/F10, Sk-Mel-5, Sk-Mel-28, and A375 were cultured in Dulbecco's modified Eagle's medium (DMEM) containing 10% fetal bovine serum, 2 mmol/L L-glutamine, 100 μmol/L non-essential amino acids, penicillin (100 U/mL), streptomycin (100 μg/mL), and 1 mmol/L sodium pyruvate (all from Sigma-Aldrich, Milan, Italy). WM3060 and WM983A were cultured in Tumor Specialized Media (1:5 Leibovitz's—MCDB153), containing 2% Inactivated FBS and 1.68 mM CaCl_2_. Cells were grown at 37°C in a humidified incubator under 5% CO_2_. The cell line PES 43 was isolated from a lung metastases of a patient from the National Cancer Institute, G. Pascale Foundation (Scala et al., 2006) and cultured in Iscove's modified Dulbecco's medium (Cambrex Bioscience, Verviers, Belgium) supplemented with heat-inactivated 10% fetal bovine serum, penicillin, and streptomycin (100 units/mL each). ADA was diluted in DMSO to produce a stock solution of 10 mM for *in vitro* experiments. All cell lines used in this study were characterized by the cell bank were they were purchased.

### Proliferation assay

Cell proliferation was measured by the 3-[4,5-dimethyltiazol-2yl]-2,5 diphenyl tetrazolium bromide (MTT) assay. The melanoma cells (A375, SK-Mel-5, SK-Mel-28, PES43, B16/F10, WM3060, and WM983A) and the NHEM cells were seeded on 96-well plates (1 × 10^4^ cells/well) and treated with *ADA* or with the other compounds: Propionyl deacylasadisulfide (PDA); methoxylatifolone (MEF); foetisulfide A (FSA); arachyl deacylasadisulfide (ARDA); deacylasadisulfide (DA)]; (10-30-100 μM) for 24–48–72 h before adding 25 μl of MTT (Sigma, Milan, Italy) (5 mg/ml in saline). Cells were incubated for additional 3 h at 37°C. Thereafter, cells were lysed and dark blue crystals were solubilized with a solution containing 50% (vol/vol) *N*,*N*-dimethyl formamide, 20% (wt/vol) sodium dodecylsulfate with an adjusted pH of 4.5. The OD of each well was measured with a microplate spectrophotometer (TitertekMultiskan MCC/340) equipped with a 620-nm filter.

### Amperometric measurement of hydrogen sulfide realease from ADA

The characterization of the potential H_2_S-generating properties of the tested compound *ADA* has been carried out by an amperometric approach, through the Apollo-4000 free radical analyzer (WPI) detector and H_2_S-selective mini-electrodes. The experiments have been carried out at room temperature (20°C). Following the manufacturer's instructions, a “PBS buffer 10x” was prepared (NaH_2_PO_4_·H_2_O 1.28 g, Na_2_HPO_4_.12H_2_O 5.97 g, NaCl 43.88 g in 500 ml H_2_O) and stocked at 4°C. Immediately before the experiments, the “PBS buffer 10x” was diluted using distilled water (1:10) to obtain the assay buffer and the pH adjusted to 7.4. The H_2_S-selective mini-electrode was equilibrated in 10 ml of the assay buffer, until the recovery of a stable baseline. Then, 100 μl of a DMSO solution of *ADA* was added (the final concentration of the tested compound was 100 μM; the final concentration of DMSO in the assay buffer was 1%). The eventual generation of H_2_S was observed for 20 min. Preliminary experiments demonstrated that DMSO 1% did not produce any interference on the amperometric recording. When required by the experimental protocol, the nucleophil L-cysteine (4 mM) was added 10 min before the addition of *ADA*. L-Cysteine did not produce any amperometric response. The correct relationship between the amperometric currents (recorded in pA) and the corresponding concentrations of H_2_S was previously determined by suitable calibration curves, which were obtained by the use of increasing concentrations of NaHS (1, 3, 5, and 10 μM) at pH 4.0.

### Flow cytometry

Apoptosis was detected with an Annexin V-FITC kit purchased from BD Pharmingen (San Diego, CA, USA) according to the manufacturer's instructions. PES 43 cells were seeded in 35 mm culture dishes and allowed to attach overnight. The cells were treated with *ADA* (100 μM) for 24–48–72 h, collected, and washed twice with PBS. Samples were then taken to determine baseline and drug-induced apoptosis by Annexin V-FITC/Propidium Iodide (PI) (Beckman Coulter; Brea, CA) double staining or PI staining and flow cytometry analysis using a FACSCanto II 6-color flow cytometer (Becton Biosciences, San Jose, CA), as described previously (Ianaro et al., 2009). To detect early and late apoptosis, both adherent and floating cells were harvested together and resuspended in annexin V binding buffer (10 mM HEPES/NaOH pH 7.4, 140 mM NaCl, 2.5 mM CaCl_2_) at a concentration of 10^6^ cells/mL. Subsequently, 5 μL of FITC-conjugated Annexin V and 5 μL of PI were added to 100 μL of the cell suspension (10^5^ cells). The cells were incubated for 15 min at room temperature in the dark. Finally, 400 μL of annexin V binding buffer was added to each tube. A minimum of 50000 events for each sample were collected and data were analyzed using FacsDiva software (Becton Biosciences).

### Preparation of cellular extracts and western blot analysis

PES 43 and A375 cells were treated with *ADA* 100 μM for 15–30–60'or for 3–6–24–48 h. Whole-cell or nuclear extracts were prepared as previously described (Ialenti et al., 2005; Panza et al., 2016). The protein concentration was measured by the Bradford method (Bio-Rad, Milan, Italy). Equal amounts of protein (40 μg/sample) from whole or nuclear cell extracts were separated by sodium dodecylsulfate polyacrylamide gel electrophoresis (SDS-PAGE) and blotted onto a nitrocellulose membranes (Trans-Blot Turbo Transfer Starter System, Biorad). The membranes were blocked for 2 h in 5% low-fat milk in PBS with 0.1% Tween 20 (PBST) at room temperature. Then the filters were incubated with the following primary antibodies: IκBα (sc-1643 Cruz Biotechnology, Santa Cruz, CA; diluted 1:200); Bcl-2 (2876, Cell Signaling, USA; diluted 1:1000), caspase 3 (9662, Cell Signaling, USA; diluted 1:1000), PARP (9542, Cell Signaling, USA; diluted 1:1000), p44/42 MAPK (Erk1/2) (9102, Cell Signaling, USA; diluted 1:1000), Phospho-p44/42 Erk MAPK (Erk1/2, Thr202/Tyr204) XP (4370, Cell Signaling, USA; diluted 1:2000), AKT (9272, Cell Signaling, USA; diluted 1:1000), Phospho-AKT (Ser473) XP (4060, Cell Signaling, USA; diluted 1:2000), c-FLIP (06-864, Millipore; diluted 1 μg/ml); XIAP (R&D System, Minneapolis; 1 μg/ml); NF-κB p65 (F-6): (sc-8008 Santa Cruz Biotechnology, Santa Cruz, CA; diluted 1:200); β-actin (Santa Cruz Biotechnology, Santa Cruz, CA; diluted 1:1000), GAPDH (2118, Cell Signaling, USA; diluted 1:1000) overnight at 4°C. The membranes were washed 3 times with PBST and then incubated with horseradish peroxidase-conjugated antibodies (Santa Cruz Biotechnology, Santa Cruz, CA; diluted 1:2000) for 2 h at room temperature. The immune complexes were visualized by the ECL chemiluminescence method and acquired by the Image Quant 400 system (GE Healthcare).

### Invasion assay

The assay was performed using chambers with polycarbonate filters with 8-μm nominal pore size (Millipore, USA) coated on the upper side with Matrigel (Becton Dickinson Labware, USA) as previously described (Ivanov et al., 2003). Briefly, the chambers were placed into a 24-well plate and melanoma cells (2.5 × 10^5^/mL) were plated in the upper chamber, with or without *ADA* (10–30 μM), in serum-free DMEM. After the incubation period (16 h), the filter was removed, and non-migrant cells on the upper side of the filter were detached with the use of a cotton swab. Filters were fixed with 4% formaldehyde for 15 min, and cells located in the lower filter were stained with 0.1% crystal violet for 20 min and then washed with PBS. The filters were examined microscopically and cellular invasion was determined by counting the number of stained cells on each filter in at least 4–5 randomly selected fields. Resultant data are presented as a mean of invaded cells ± SEM /microscopic field of three independent experiments.

### Animals

The experimental procedures, according to Italian (DL 26/2014) and European (n. 63/2010/UE) regulations on the protection of animals used for experimental and other scientific purposes, were approved by the Italian Ministry. All studies involving animals are reported in accordance with the ARRIVE guidelines for reporting experiments involving animals (Kilkenny et al., 2010; McGrath et al., 2010). Mice were observed daily and humanely euthanized by CO_2_ inhalation if a solitary subcutaneous tumor exceeded 1.5 cm in diameter or mice showed signs referable to metastatic cancer. All efforts were made to minimize suffering. Female C57BL/6 mice (18−20 g) were purchased from Charles River Laboratories, Inc. Mice were housed at the Animal Research Facility of the Department of Pharmacy of the University of Naples Federico II.

### Tumor metastasis assay

B16/F10 (5 × 10^5^) murine melanoma cells were collected in PBS and injected via tail vein into syngeneic C57BL/6J mice. The mice were equally randomized into three groups (8 mice/group): 0.9% normal saline control group, *ADA* 5 mg·kg^−1^ group, *ADA* 50 mg·kg^−1^ group. Starting on the 1st day after tumor cell inoculation, test compounds were given orally every day for 14 days. Then the mice were weighed and sacrificed. The lungs were removed and washed with PBS. The percentage of metastatic area was calculated using ImageJ software (ImageJ).

### Data analysis

Data are expressed as mean ± SEM of *n* experiments. Data were analyzed and presented using GRAPHPAD PRISM software (GraphPad). Significance was determined using Student's 2-tailed *t*-test. Results were considered significant at *P*-values ≤ 0.05 and are labeled with a single asterisk. In addition, *P*-values ≤ 0.01 and 0.001 are designated with double and triple asterisks, respectively.

## Results

### *ADA* suppresses the proliferation of human melanoma cells *in Vitro*

**Figure 1:**
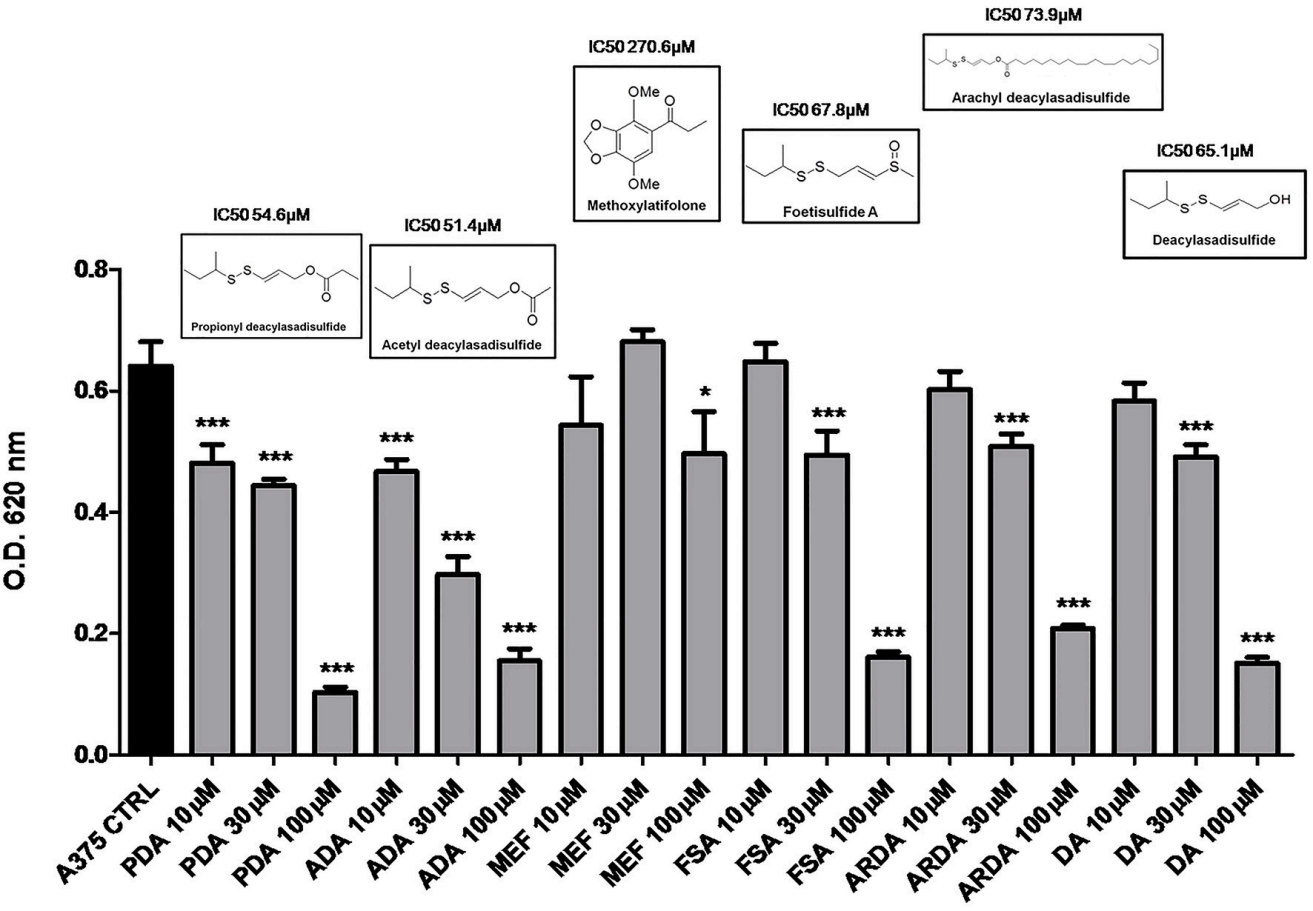
**Disulfide compounds from asafetida inhibit the proliferation of human melanoma cells A375**. A375 cells were treated with increasing concentration (10–30–100 μM) of several disulfide compounds (propionyl deacylasadisulfide, PDA; acetyl deacylasadisulfide, ADA; foetisulfide A, FSA; arachyl deacylasadisulfide, ARDA; deacylasadisulfide, DA) and one no sulfur-containing compound (methoxylatifolone, MEF). Growth inhibition was measured at 72 h using the MTT assay and is expressed as OD values. All the compounds tested, with the only exception of the MEF, inhibited the proliferation of melanoma cells although with different potency. Experiments were run in triplicate, each performed in quadruplicate (^*^P < *0.05;* ^***^P < *0.001* vs. control [CTRL]).

In preliminary experiments, the disulfide compounds obtained from asafoetida were evaluated for their anti-proliferative activity on human melanoma cells A375 (Figure 1). After an initial screening, we found that all the sulfur-containing compounds *ADA*, PDA, FSA, ARDA, and DA inhibited the proliferation of melanoma cells although with different potency (Figure 1). On the other hand, the structurally unrelated MEF was practically inactive. Therefore, the disulfide group is essential for the anti-proliferative effect. Among the active compounds *ADA* and PDA showed similar activity on the human melanoma cell line A375 (IC_50_ 51.4 and 54.6 μM, respectively). The selection of *ADA* for further investigation was based on the relative abundance of this metabolite in the plant, the higher availability for pharmacological evaluation as well as the presence of a clear concentration-related effect as compared to PDA.

*ADA* also inhibited the proliferation of all the other human melanoma cell lines employed in this study, namely SK-Mel-5, SK-Mel-28, WM983A, and PES43 cell lines, all carrying the BRAF^v600E^ mutation and the WM3060 cell line wild type for BRAF, without affecting NHEM proliferation (Table 1). A marked inhibition of cell proliferation was observed also in the murine cell line B16/F10 after incubation with increasing concentration of *ADA* (Table 1). Among the human cell lines employed the high metastatic PES43 and A375 were the most sensitive to the anti-proliferative activity of *ADA*. In fact, as shown in Table 1 increasing concentration of *ADA* (10-30-100 μM) inhibited PES43 proliferation at 72 h by 38, 61, and 71%, respectively (*P* < 0.001, *n* = 5) and A375 cell proliferation by 28, 54, and 76% respectively (*P* < 0.001, *n* = 5). Thus, these cell lines were selected for the molecular studies.

**Table 1:** **Anti-proliferative effect of ***ADA*** on different human melanoma cell lines**.

**Cell line**	**CTRL**	**ADA 10 μM**	**ADA 30 μM**	**ADA 100 μM**
NHEM	0.230 ± 0.01	0.233 ± 0.002	0.223 ± 0.003	0.219 ± 0.01
PES43	0.532 ± 0.05	0.329 ± 0.03^***^	0.208 ± 0.008^***^	0.154 ± 0.003^***^
A375	0.641 ± 0.02	0.467 ± 0.02^***^	0.297 ± 0.03^***^	0.155 ± 0.002^***^
Sk-Mel-28	0.520 ± 0.01	0.504 ± 0.02	0.485 ± 0.02	0.115 ± 0.002^***^
Sk-Mel-5	0.309 ± 0.007	0.291 ± 0.001	0.252 ± 0.01	0.07 ± 001^***^
WM983A	0.218 ± 0.002	0.201 ± 0.005	0.195 ± 0.003	0.093 ± 0.002^***^
WM3060	0.223 ± 0.003	0.190 ± 0.004	0.173 ± 0.004^**^	0.118 ± 0.002^***^
B16F10	1.0 ± 0.01	0.757 ± 0.01^***^	0.41 ± 0.005^***^	0.101 ± 0.001^***^

NHEM, A375, Sk-Mel-5, Sk-Mel-28, PES43, WM3060, WM968A, and B16/F10 cells were treated with increasing concentration (10-30-100 μM) of ADA. Growth inhibition was measured at 72 h using the MTT assay and is expressed as OD values ± SEM. Experiments were run in triplicate, each performed in quadruplicate (

** P < 0.01;

*** *P < 0.001 vs. control [CTRL])*.

### *ADA* induces apoptosis and activation of caspase-3 in human melanoma cells

**Figure 2:**
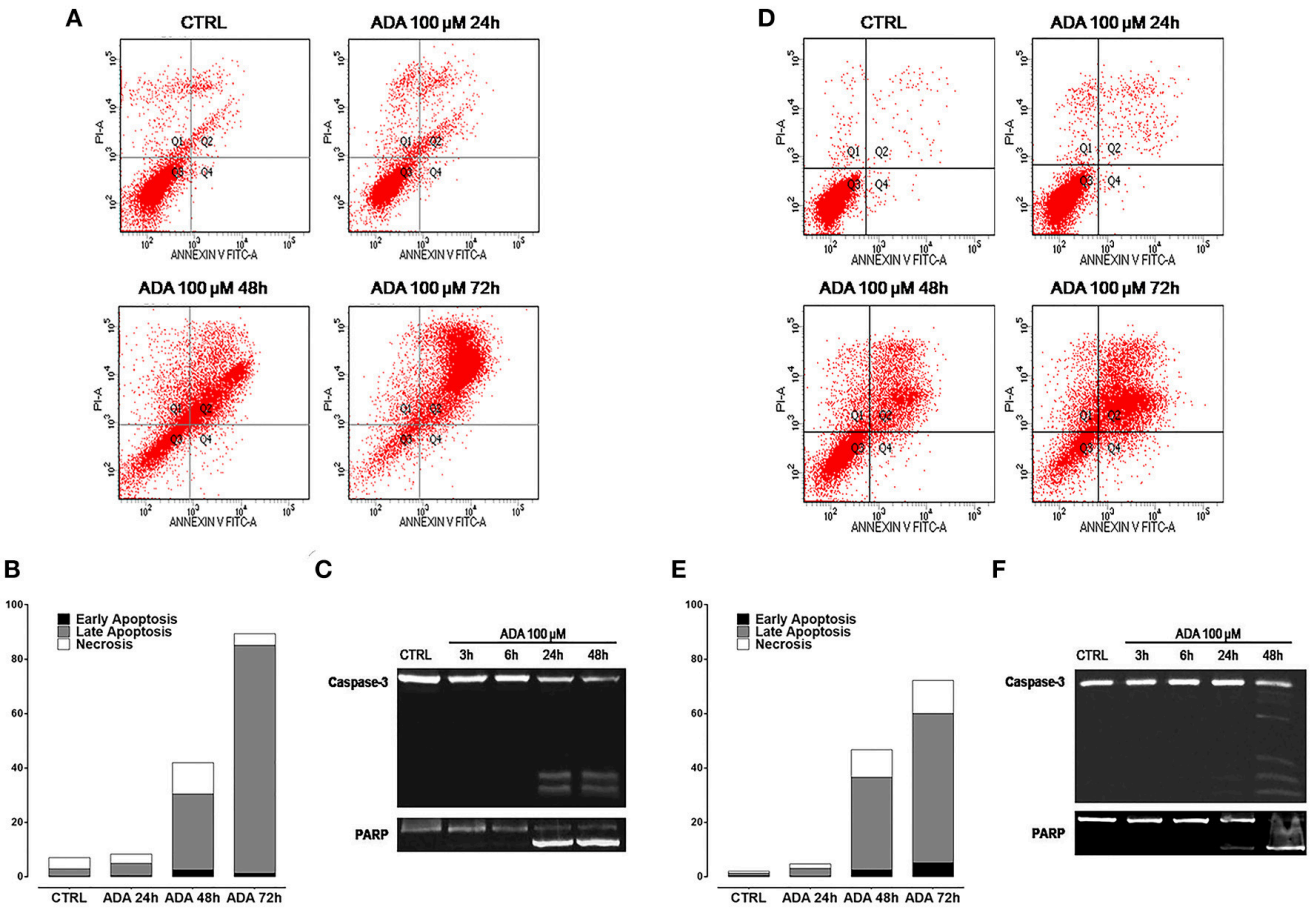
*****ADA*** induces apoptosis in metastatic melanoma cells PES43 and A375**. PES43 and A375 cells were treated with *ADA* (100 μM) at different time points and apoptosis was determined by flow cytometry analysis. Apoptosis was determined by annexin V/propidium iodide (PI) staining, which detects the externalization of phosphatidylserine (PS). This dual staining distinguishes between unaffected cells (unlabeled; quadrant 3, Q3), early apoptotic cells (annexin V positive; quadrant 4, Q4), late apoptotic cells (annexin V positive, PI positive; quadrant 2, Q2), and necrotic (PI positive; quadrant 1, Q1). Treatment of PES43 cells **(A)** or A375 cells **(D)** for 24–48–72 h with *ADA* (100 μM) resulted in a time-dependent induction of apoptosis. **(B,E)** Quantitative analysis of apoptosis at various time points showing that at 72 h about 80% of PES43 cells **(B)** or A375 cells **(E)** treated with *ADA* exhibit markers of late apoptosis. Experiments (*n* = 5) were run in triplicate. **(C,F)** Western blot analysis of caspase 3 and PARP in PES43 **(C)** or A375 cells **(F)** whole-cell lysates. PES43 or A375 cells were incubated with *ADA* 100 μM at different time points and at 24 and 48 h cleavage of caspase 3 and of its substrate PARP was observed.

To verify if the anti-proliferative effect of *ADA* was related to its ability to induce apoptosis of cancer cells, the cytofluorimetric assay, with Annexin V/PI dual staining, was carried out on melanoma cells PES43 and A375. This dual staining distinguishes between unaffected (unlabeled; quadrant 3, Q3), early apoptotic (Annexin V positive; quadrant 4, Q4), late apoptotic (Annexin V positive, PI positive; quadrant 2, Q2), and necrotic (PI positive; quadrant 1, Q1) cells. Treatment of PES 43 and A375 cells for 24–48–72 h with *ADA* (100 μM) resulted in a significant and time–dependent induction of apoptosis (Figures 2A,B,D,E). The pro-apoptotic effect of *ADA* was confirmed by a time-dependent cleavage of caspase 3, the main effector caspase, and of its substrate poly (adenosine diphosphate-ribose) polymerase (PARP) (Figures 2C,F).

### *ADA* inhibits NF-κB activation and down-regulates NF-κB dependent anti-apoptotic genes

**Figure 3:**
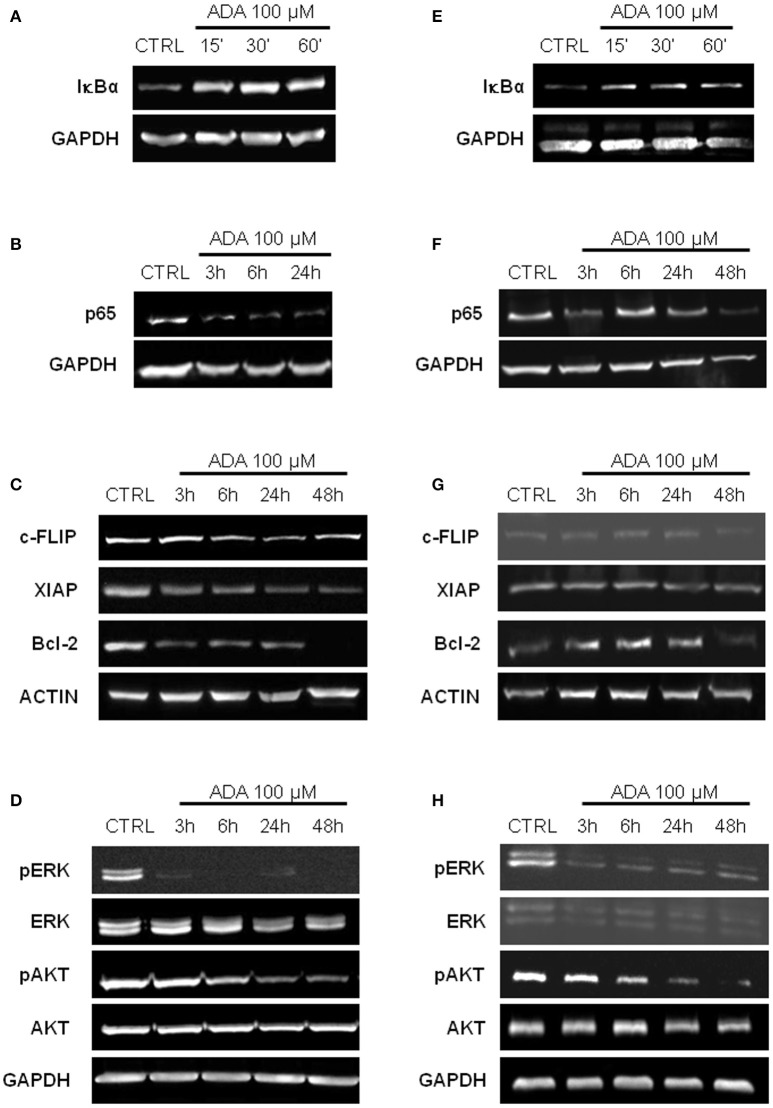
*****ADA*** inhibits NF-κB activation and down-regulates MAPK/ERK and PI3K/AKT pathways in metastatic melanoma cells**. Western blot analysis carried out on the whole-cell extracts obtained from PES43 **(A)** and A375 **(E)** cells treated with *ADA* 100 μM shows an inhibition of IκBα degradation at 30 and 60 min. Nuclear extracts from control and *ADA*-treated PES43 **(B)** and A375 **(F)** cells collected at 3–6–24–48 h were analyzed by Western blot for NF-κB activation as p65 nuclear translocation. Both cell lines displayed a constitutively high expression of p65 into the nucleus that was reduced by *ADA*. Western blot analysis of c-FLIP, XIAP, and Bcl-2 carried out on PES43 **(C)** and A375 **(G)** cells treated with *ADA* 100 μM for 3–6–24–48 h. *ADA* decreased the expression of all the anti-apoptotic genes analyzed in both cell lines. Actin and GAPDH were detected as loading control. Experiments (*n* = 5) were run in triplicate. Western blot analysis of phospho- and total Akt and phospho- and total ERK in PES43 **(D)** and A375 **(H)** cells treated with *ADA* (100 μM) for 3–6–24–48 h. Both phospho-AKT (p-AKT) and phospho-ERK (p-ERK) band intensity was time-dependently reduced following treatment with *ADA* (100 μM). GAPDH was detected as a loading control. Experiments (*n* = 5) were run in triplicate.

It has been reported that in melanoma constitutive activation of NF-κB confers tumor survival capacity and apoptosis avoidance (Ueda and Richmond, 2006). In order to analyze if the inhibition of apoptosis induced by *ADA* was involving the NF-κB pathway, PES 43 and A375 cells were treated with *ADA* 100 μM for 15, 30, and 60 min and western blot analysis was carried out on cellular extracts. As shown in Figures 3A,E an inhibition of IκBα degradation at the earliest time points following *ADA* incubation was observed. This effect was abided by the inhibition of NF-κB nuclear translocation as demonstrated by a reduction in band intensity of the subunit p65 (Figures 3B,F). Finally, western blot analysis showed that *ADA* markedly decreased the expression of three anti-apoptotic proteins whose expression is modulated by the transcriptional activity of NF-κB, (XIAP, c-FLIP, and Bcl-2) confirming NF-κB involvement (Figures 3C,G).

### Effect of *ADA* on MAPK/ERK and PI3/AKT pathways

The Mitogen-Activated Protein Kinase (MAPK)/ERK and the Phosphoinositide 3-Kinase (PI3K)/AKT pathways are two of the most frequently deregulated pathways in melanoma (Hodis et al., 2012). They play an important role in melanoma development and progression and are involved in the mechanism of resistance to targeted therapy (Flaherty et al., 2012). As shown in Figures 3D,H, both phospho-AKT (p-AKT) and phospho-ERK (p-ERK) band intensity was time-dependently reduced following treatment with *ADA* (100 μM).

### Effect of *ADA* on cells invasion

**Figure 4:**
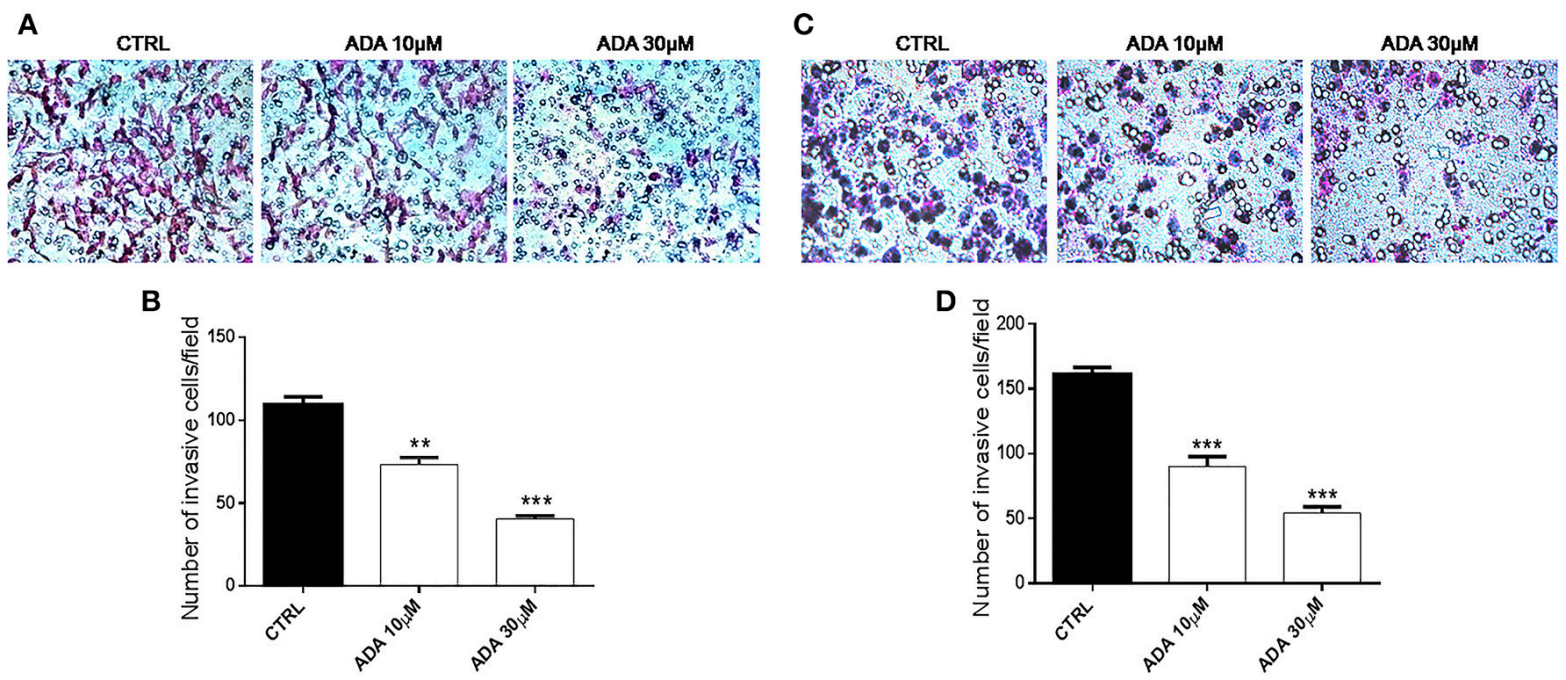
*****ADA*** inhibits the invasion of metastatic melanoma cells**. *ADA* (10–30 μM) suppresses PES43 and A375 cell invasion as measured by a transwell cell invasion assay. Representative field of invasive cells PES43 **(A)** and A375 **(C)** on the membrane Average number of invasive cells PES43 **(B)** and A375 **(D)** from triplicate measurements. (^**^P < *0.01;* ^***^P < *0.001* vs. control [CTRL]).

To determine whether *ADA* affected the invasive ability of the metastatic melanoma cells PES 43 and A375, we performed cell invasion assays using a transwell system. As shown in Figures 4A,B, *ADA* at 10 and 30 μM significantly inhibited the invasiveness of PES43 cell line by 33 and 63%, respectively (*P* < 0.01; *P* < 0.001 vs. control; *n* = 5). *ADA* (10–30 μM) produced a comparable effect on A375 cell line (Figures 4C,D) inhibiting the invasiveness by 45 and 67% respectively (*P* < 0.001 vs. control; *n* = 5).

### *ADA* inhibits metastatic melanoma *In vivo* in mice

**Figure 5:**
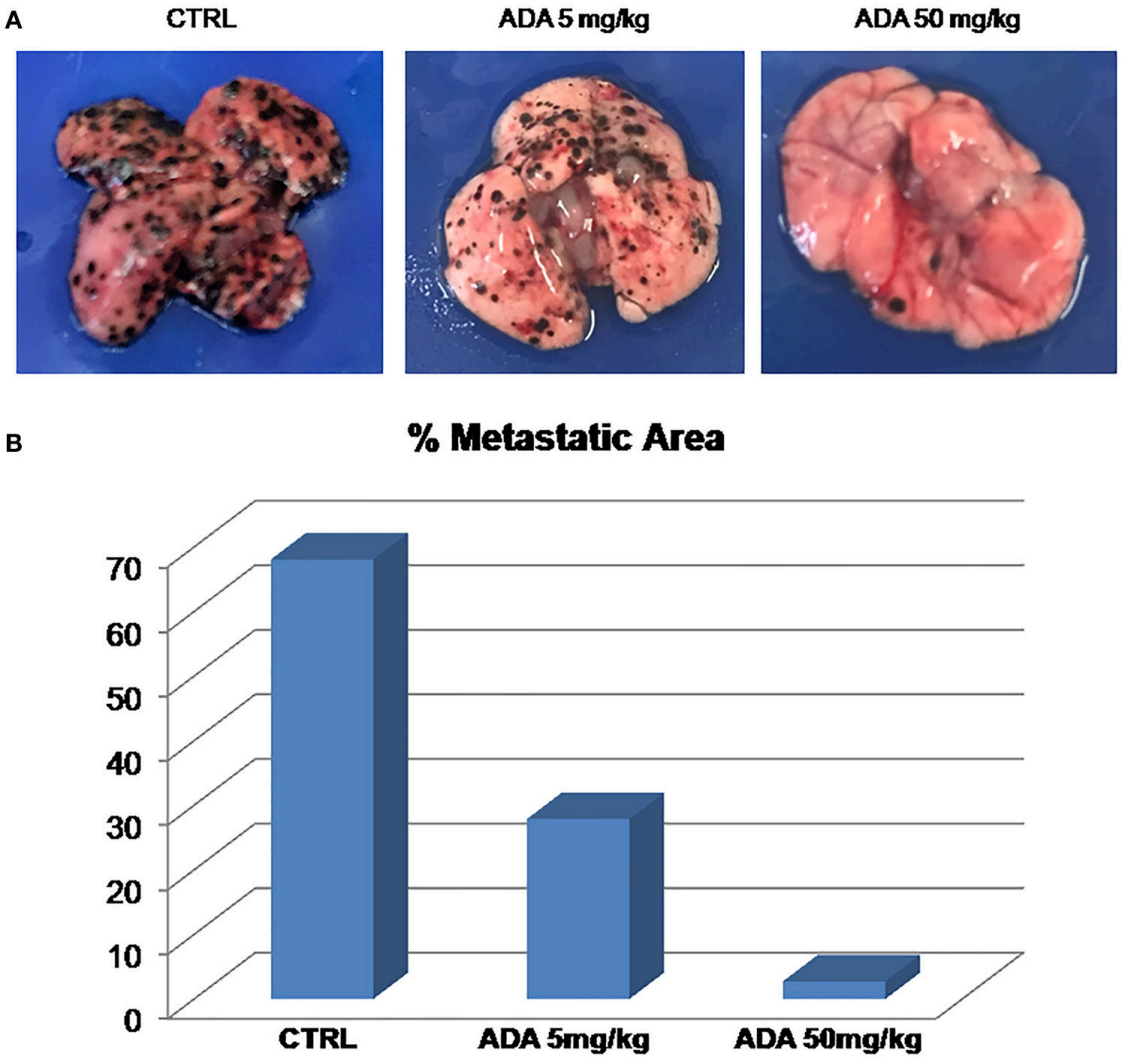
*****ADA*** inhibits B16 melanoma metastasis ***in vivo*****. B16-F10 mouse melanoma cells were injected through the lateral tail vein of C57BL/6J mice (*n* = 8 per group). *ADA* (5 or 50 mg·kg^−1^) was given orally to mice; control mice received vehicle only. *ADA* 5 and 50 mg·kg^−1^ reduced the metastatic area by 60 and 96% respectively. **(A)** Representative macroscopic pictures of mouse lungs, 14 days after inoculation. **(B)** Histogram with the percentage of metastatic affected area.

To properly investigate on the role of *ADA* on melanoma progression, we exploited a murine model of metastatic melanoma induced following the injection of the murine melanoma cells B16/F-10 into the tail vein of C57BL/6J mice. Groups of mice were treated with different doses of *ADA* (5 and 50 mg·kg^−1^, p.o.) while control group received the vehicle only. Fourteen days after tumor implant, lungs were removed and the percentage of metastatic affected area was calculated by using the software Image J. As shown in Figures 5A,B, *ADA* (5–50 mg·kg^−1^) caused a dose-dependent reduction of lung metastases. The higher dose tested e.g., 50 mg·kg^−1^ reduced the total area of about 96% (2.74% vs. control 68.1%).

### *ADA* releases hydrogen sulfide

**Figure 6:**
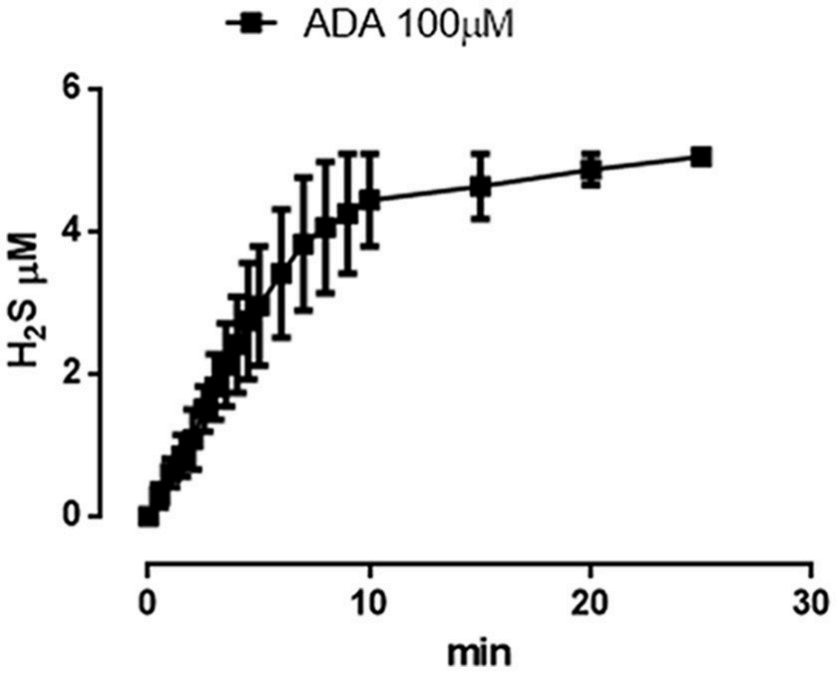
**ADA releases hydrogen sulfide**. Curve describes the increase of H_2_S concentration, with respect to time, following the incubation of *ADA* (100 μM) in the assay buffer in the presence of the nucleophile l-cysteine. H_2_S was recorded by amperometry; the vertical bars indicate the SEM.

The incubation of *ADA* 100 μM in aqueous solution in presence of the nucleophil L-cysteine led to the generation of a time-related increase in H_2_S concentration. The rise of the H_2_S production reached a steady-state after about 10 min and the highest concentration of H_2_S, recorded after 20 min was 5.05 ± 0.04 μM (Figure 6).

## Discussion

In the present study, we describe for the first time, the anti-cancer properties of *ADA*, a naturally occurring H_2_S donor, isolated and purified from *Ferula assa-foetida L*. Sulfur compounds contained within vegetables may be chemically or enzymatically transformed in the human body with subsequent formation of H_2_S (Jacob et al., 2008) and their consumption has been associated to chemopreventive effects (Krasilnikov et al., 2003). In particular, the garlic-derived organic polysulfide DATS has been shown to exhibit anticancer activity (Wang et al., 2012). Furthermore, epidemiological studies have shown that people assuming a diet rich in cruciferous vegetables (i.e., broccoli and cabbage) have a minor incidence of breast, lung, prostate, colon, and bladder cancer (Spitz et al., 2000; Ambrosone et al., 2004; Tang et al., 2008; Traka et al., 2008). This activity has been mainly ascribed to the isothiocyanate component abundantly present in these vegetables. Phytochemicals rich in sulfur, in particular diet-derived compounds, have therefore been proposed and applied in clinical trials as cancer chemopreventive/chemotherapeutic agents. These results along with studies performed using inhibitors of the enzymes responsible of H_2_S production have led to hypothesize a role for this metabolic pathway in cancer as recently reviewed (Szabo, 2016). We have previously established that sulfurated compounds can inhibit human melanoma cell proliferation (Panza et al., 2015). Here we describe the anti-tumoral activity of a newly identified natural H_2_S-donor from *Ferula assa-foetida L*. Interestingly, among all the compounds screened only the non-sulfur containing methoxylatifolone resulted inactive. This evidence further support our working hypothesis that the presence of sulfur is required in order to display anti-proliferative activity in melanoma. Indeed, all the sulfur containing compounds isolated were active on A375 melanoma cells and, among these, *ADA* and PDA showed the highest potency. *ADA* was selected to perform the study for the following reasons: (i) it displayed a clear concentration-dependent effect, as opposite to PDA that appeared to have a threshold concentration; (ii) it was the most abundant vinyl disulfide present in *Ferula assa-foetida*. The latter aspect has to be outlined since the abundance of a purified natural compound makes it possible to perform a more in deep and accurate pharmacological evaluation of its biological activities. Thus, we further investigated on *ADA* activity by using a panel of melanoma cell lines. Among the human cells tested, the metastatic cell lines PES 43 and A375 resulted the most sensible to the anti-proliferative activity of *ADA* and thus were selected for the mechanistic studies. Since in many cases the anti-proliferative effect of H_2_S-releasing molecules is related to their ability to activate the apoptotic machinery we investigated on this biochemical event performing flow citometry and western blot analysis on both cell lines. The results confirmed that *ADA* anti-proliferative effect relies on induction of apoptosis. This was confirmed by the cytofluorimetric assay with Annexin V/PI dual staining and by the time-dependent cleavage of caspase 3 and of its substrate PARP. In order to better understand the mechanism(s) underlying the apoptotic effect we investigated on the downstream signaling, looking at a possible involvement of NF-κB. Indeed, this pleiotropic transcription factor plays important roles in controlling cell proliferation, apoptosis, and oncogenesis. NF-κB activation has been also shown to promote melanoma tumor progression (Richmond, 2002; Ivanov et al., 2003). In fact, in late melanoma stage, NF-κB is up-regulated and inhibits cell apoptosis by enhancing the expression of several anti-apoptotic genes such as XIAP (Deveraux et al., 1998), c-FLIP (Micheau et al., 2001), and BclxL genes (Ravi et al., 2001). In most non-transformed cell types, NF-κB complexes bound to an inhibitor, IκB, are largely cytoplasmic. On activation, the IκB proteins become phosphorylated, ubiquitinated, and subsequently degraded. Freed NF-κB accumulates in the nucleus, where it enhances the transcription of specific genes. We demonstrated, by western blot analysis, that *ADA* inhibited both the phosphorylation and degradation of IkBα thereby preventing the nuclear translocation of the p65 subunit containing the transcriptional activation domain. The involvement of NF-κB was further supported by the finding that the expression of the anti-apoptotic genes c-FLIP, XIAP and Bcl-2 was also reduced. Indeed, all these three genes are transcriptionally regulated by NF-κB. As well as for NF-κB also the serine/threonine kinase Akt is constitutively activated in human melanomas (Madhunapantula et al., 2011). Although it may not be essential for initiation of melanoma, Akt activation facilitates melanoma progression by enhancing cell survival through up-regulation of NF-κB (Dhawan et al., 2002). Our results clearly show that *ADA* down-regulates Akt activity by reducing the phosphorylation on Ser473. Therefore, *ADA* inhibits the constitutive activated PI3K/Akt pathway and its downstream target NF-κB in melanoma cells.

Another key player involved in melanoma progression and resistance to current therapies is the Ras/Raf/MAPK pathway that is strictly linked to Akt pathway (Dhillon et al., 2007; Sullivan and Flaherty, 2013). In fact, several new drugs interfering with these two pathways are in advanced clinical phase for the treatment of resistant metastatic melanoma. Our results demonstrated an inhibitory effect of *ADA* also on the Ras/Raf/MAPK pathway. In fact, following incubation of melanoma cells with *ADA*, we observed a reduction of the phosphorylation and activation of ERK1/2. The dual inhibitory effect exhibited by *ADA* on both activating pathways is in line with the therapeutic strategy that are currently clinically explored in melanoma. It is well-known that mutant B-RAF may indirectly activate NF-κB through constitutive activation of the downstream effector protein ERK1/2 and the up-regulation of inflammatory cytokines leading to increases in survival, proliferation and invasiveness (Norris and Baldwin, 1999). In particular the increased ability of cancer cells to invade adjacent tissues is one of the critical steps leading to metastasis (Chaffer and Weinberg, 2011). This process is poorly understood even if it is the major cause of cancer-related mortality. Therefore, many investigators are trying to find strategies to suppress tumor growth as well as tumor metastasis. We addressed this particular issue by performing *in vitro* and *in vivo* experiments. Firstly, we demonstrated that *ADA* inhibits melanoma cells invasive capability *in vitro* by almost 70% at the higher dose used. All these *in vitro* evidence clearly indicated that ADA, by interfering with more than one mechanism involved in melanoma progression and spreading, has the potential to act also *in vivo*. In order to address this issue we used a model of pulmonary metastasis in mice that mimic the common and virulent phenomena of human metastasis and is widely used for the pre-clinical evaluation of drugs (Overwijk and Restifo, 2001). The injection of *ADA* to mice significantly suppressed (about 96% as compared to untreated mice) metastatic tumor growth in the lung suggesting that ADA reduced migration and growth of melanoma cancer cells in lung tissue. Of particular interest is the fact that the anti-tumor effect displayed by *ADA* is more sustained of that displayed by the well-characterized H_2_S-releasing molecule DATS (Panza et al., 2015).

It is necessary to point out that B16-F10, as any tumor cells, once injected intravenously migrate into the lungs. Therefore, the term pulmonary metastasis is widely used even though every resulting pulmonary nodule is technically an independent “primary” tumor rather than a true metastasis (Overwijk and Restifo, 2001). Nevertheless, within the limit of this preclinical model, *ADA* also *in vivo* displayed a significant and marked anticancer activity.

A recent investigation has reported a detailed picture of the sulfur-containing compounds of asafoetida. These mixed *S*-alkyl-*S*-alkenyldisulfides are able to release H_2_S due to their electrophilic nature and tendency to react with different nucleophilic (Nu:) agents (Shokoohinia et al., 2013).

In conclusion, we have established that *ADA*, a sulfur-containing compound of asafetida, not only suppresses melanoma cell growth and tumor cell migration *in vitro* but it is also active *in vivo*. Metastasis in cancer patients is associated with poor prognosis and death. Therefore, the finding of new strategies to suppress tumor growth and tumor metastasis is an unmet clinical need. The results of our study point toward this aim indicating *ADA* as a lead compound for the development of a new class of drugs active in metastatic melanoma based on the presence of labile sulfur in their structure.

## Author contributions

PD and EP were responsible for acquisition, analysis and interpretation of data, and redaction of the manuscript. CA, GE, RC, and VC carried out acquisition, analysis, and interpretation of data. OT, YS and GP were responsible for the critical reading of the manuscript. GC was responsible for interpretation of data and critical reading of the manuscript. AI performed conception and design, analysis and interpretation of data, and redaction of the manuscript. All authors read and approved the final manuscript.

## Funding

Funding of this research was provided by the Italian Government grants (PRIN 2012 no: 2012WBSSY4_005).

### Conflict of interest statement

The authors declare that the research was conducted in the absence of any commercial or financial relationships that could be construed as a potential conflict of interest.

## Abbreviations

H_2_S: hydrogen sulfide
ADA: acetyl deacylasadisulfide
PDA: propionyl deacylasadisulfide
FSA: foetisulfide A
ARDA: arachyl deacylasadisulfide
DA: deacylasadisulfide
MEF: methoxylatifolone.
